# Teaching Hands-On Informatics Skills to Future Health Informaticians: A Competency Framework Proposal and Analysis of Health Care Informatics Curricula

**DOI:** 10.2196/15748

**Published:** 2020-01-21

**Authors:** A Hasan Sapci, H Aylin Sapci

**Affiliations:** 1 Adelphi University Garden City, NY United States; 2 Hancock, MI United States

**Keywords:** health informatics curriculum, skill-based training, hands-on health informatics training

## Abstract

**Background:**

Existing health informatics curriculum requirements mostly use a competency-based approach rather than a skill-based one.

**Objective:**

The main objective of this study was to assess the current skills training requirements in graduate health informatics curricula to evaluate graduate students’ confidence in specific health informatics skills.

**Methods:**

A quantitative cross-sectional observational study was developed to evaluate published health informatics curriculum requirements and to determine the comprehensive health informatics skill sets required in a research university in New York, United States. In addition, a questionnaire to assess students’ confidence about specific health informatics skills was developed and sent to all enrolled and graduated Master of Science students in a health informatics program.

**Results:**

The evaluation was performed in a graduate health informatics program, and analysis of the students’ self-assessments questionnaire showed that 79.4% (81/102) of participants were not confident (not at all confident or slightly confident) about developing an artificial intelligence app, 58.8% (60/102) were not confident about designing and developing databases, and 54.9% (56/102) were not confident about evaluating privacy and security infrastructure. Less than one-third of students (24/105, 23.5%) were confident (extremely confident and very confident) that they could evaluate the use of data capture technologies and develop mobile health informatics apps (10/102, 9.8%).

**Conclusions:**

Health informatics programs should consider specialized tracks that include specific skills to meet the complex health care delivery and market demand, and specific training components should be defined for different specialties. There is a need to determine new competencies and skill sets that promote inductive and deductive reasoning from diverse and various data platforms and to develop a comprehensive curriculum framework for health informatics skills training.

## Introduction

### Background

The National Center for Education Statistics defines competency as a combination of skills, abilities, and knowledge needed to perform a specific task [1]. The 21st century health informatics jobs will require specific skills such as collecting data from wireless medical devices and integrating real-time data analytics and artificial intelligence (AI) algorithms in clinical patient monitoring apps. Even though health informatics is a distinct interdisciplinary field that provides various paths to different careers and covers a variety of topics, the specific skill sets required by different employers vary owing to the increasing rate of technological developments [2]. In addition, the skills needed for health informaticians vary significantly depending on the position [3], and health informatics students need skills pertinent to their professional experience for their future career paths [4]. However, there are still significant gaps in workforce skills training, and studies examining students’ perspectives on required skill sets are limited. As academicians, students, employers, and people working in the health care industry have different perspectives and priorities for required informatics skills, identifying health informatics skill sets for graduate students has always been a challenge. Although students with a clinical background might need mobile health (mHealth) skills to diagnose and treat patients, those with information technology background might need advanced technical and programming skills to design and develop patient-centered health information systems, connected medical devices, consumer-directed mHealth apps, the internet of things–linked wearable solutions and analytics solutions that utilize machine learning, and personalized medicine apps that use AI algorithms [5].

### Evolving Health Informatics Competencies and Skills Training Recommendations

The first international recommendations to develop health informatics educational activities were published by the International Medical Informatics Association (IMIA) in 2000 [6]. IMIA determined 4 knowledge or skill domains for international information technology users and biomedical and health informatics specialists: (1) methodology and technology for the processing of data, information, and knowledge in medicine and health care; (2) medicine, health and bioscience, and health system organization; (3) informatics and computer science, mathematics, and biometry; and (4) optional modules (Table 1). IMIA has been developing self-assessment requirements, pilot-testing the procedure, and conducting site visits since 2012. The organization also conducted a strengths, weaknesses, opportunities, and threats analysis for their accreditation process and determined that their educational recommendations could be used on all continents. Currently, IMIA is the only organization that develops international accreditation competencies [7-9].

**Table 1:** The evolution of curriculum requirements for health informatics programs.

Domain (International Medical Informatics Association 2000; International)	Facet (CAHIIM^a^ 2012; National)	Foundational domains (CAHIIM/American Medical Informatics Association 2017; National)
Biomedical and health informatics core knowledge and skills	I. Information systems—concerned with such issues as information systems analysis, design, implementation, and management	F1. Health
Medicine, health and biosciences, health system organization	II. Informatics—concerned with such issues as the structure, function and transfer of information, sociotechnical aspects of health computing, and human-computer interaction.	F2. Information science and technology
Informatics/computer science, mathematics, biometry	III. Information technology—concerned with such issues as computer networks, database and systems administration, security, and programming	F3. Social and behavioral science
Optional modules	IV. Additional desired course content: epidemiology; quantitative, qualitative, and mixed methods; and biomedical sciences.	F4. Health information science and technology F5. Human factors and sociotechnical systems F6. Social and behavioral aspects of health F7. Social, behavioral, and information science and technology applied to health F8. Professionalism F9. Interprofessional collaborative practice F10. Leadership

^a^CAHIIM: Commission on Accreditation for Health Informatics and Information Management Education.

With the adoption of digital technologies around the world, several countries have focused on education initiatives to improve populations’ 21st century digital skills [10]. Professional organizations have developed their country-specific health informatics competencies; for instance, the Health Informatics Society of Australia developed a competency framework for the Australian health care system [11].

As there are several international and national initiatives to develop health informatics competencies, determining the standard comprehensive health information skill sets has always been a challenging task because of the continually evolving technology. National health informatics organizations and accreditation agencies have been specifying their own standards because of the lack of universal standards. For example, Canada’s Health Informatics Association (Digital Health Canada, known as COACH before 2017) developed 51 competencies about information management, information technology, clinical/health services, Canadian Health System, organizational and behavioral management, project management, and analysis and evaluation in 2009 [12]. The Australian Health Informatics Education Council identified 45 core competencies for the Australian workforce [13]. In the United States, the Commission on Accreditation for Health Informatics and Information Management Education (CAHIIM) and the American Medical Informatics Association (AMIA) have been establishing curriculum requirements. Even though CAHIIM’s 2012 curriculum requirements did not include any skill sets training, the revised 2017 recommendations were more specific and included skill definition for 7 foundational domains (Table 1) [14].

Another notable development was the formation of the eHealth Collaboration Workforce Development Workgroup in 2013. A Web-based database about health information technology competencies to identify the gaps between information and communication technology (ICT) competency and knowledge deficiencies was built by the United States and the European Union (EU) [15]. This comprehensive database encompasses 5 domains (administration, direct patient care, engineering/information systems/ICT, and informatics and research/biomedicine) and consists of 33 competency areas including electronic health (eHealth); mHealth; telehealth; data compiling, analysis, modeling, and reporting; and clinical decision support and pathways. This project was funded by Horizon 2020, which was the EU’s most significant research program [16]. Furthermore, the EU-US eHealth Work Project, which began in September 2016, currently conducts research to map skills and competencies and develop tools. This initiative plans to publish a comprehensive set of foundational curricula and advance eHealth/health information technology workforce when they complete the project [14,15].

The accreditation process evaluates an academic institution’s effectiveness in achieving its stated mission, and the graduate education programs that participate in the voluntary accreditation process should comply with the regional, national, or independent accrediting agencies’ core curriculum requirements. This is an important process to ensure the accountability of academic training programs and is widely considered as the de facto standard for quality assessment and continuous improvement.

In the United States, although the CAHIIM accredits undergraduate and graduate health informatics programs, the American Health Information Management Association (AHIMA) and the Commission on Certification for Health Informatics and Information Management certify individuals. In 2012, CAHIIM published the curriculum requirements for a master’s in health informatics degree and 3 mandatory facets about (1) the design, analysis, implementation, and management of information systems; (2) sociotechnical aspects; human-computer interaction; and structure, function, and transfer of information; and (3) computer networks, security, programming, database, and systems administration were determined [14]. In addition, one optional facet about optional courses such as medical terminology; anatomy; physiology; quantitative, qualitative, and mixed methods; and epidemiology was recommended (Table 1) [14].

In 2017, CAHIIM revised the accreditation standards for master’s degree programs in health informatics and published a revised version of core competencies that consists of the following foundational domains: (1) health; (2) information science and technology; (3) social and behavioral science; (4) health information science and technology; (5) human factors and sociotechnical systems; (6) social and behavioral aspects of health; (7) social, behavioral, and information science and technology applied to health; (8) professionalism; (9) interprofessional collaborative practice; and (10) leadership (Table 1) [14,17,18].

After the discussion about the need to explore the description of *core informatics competencies* in 2001, the AMIA education committee proposed establishing a medical informatics certification program the following year. AMIA’s working groups have been working on the definition and description of clinical informatics subspecialty and determining core competencies for biomedical and health informatics [19], whereas the Centers for Disease Control and Prevention has been leading a similar initiative for public health informaticians [20]. AMIA joined CAHIIM in 2015 and acknowledged the need for competency descriptions in a usable form. The Health Informatics Accreditation Council also started working on the revision of CAHIIM’s Health Informatics Accreditation Standard [21]. AMIA published the core competencies for health informatics education as an organizational member. Table 2 lists the foundational domains that list skills in CAHIIM’s revised skill recommendations document [22]. This new skills framework consists of various competency titles related to the leadership; professionalism; interprofessional collaborative practice; social, behavioral, and information science; social and behavioral aspects of health; human factors; and health information science and technology foundational domains, but it does not provide specific details.

New job opportunities for health informatics professionals require specific skill sets to utilize new cutting-edge, patient-focused delivery tools. Eligibility requirements for an advanced health informatics certification were proposed in 2016 [23]. Following this proposal, AMIA conducted the first informatics workforce survey in 2017 to build an inventory of informaticians’ unique skills and knowledge in the United States. The workforce survey evaluated professionals’ and students’ opinions on pursuing professional credentials and essential tasks in their informatics work [24]. Similarly, Digital Health Canada has conducted several competency surveys in Canada [25].

**Table 2:** The Commission on Accreditation for Health Informatics and Information Management Education’s revised health informatics skills according to the American Medical Informatics Association 2017 core competencies.

Foundational domains	Skills
F4. Health information science and technology	Design a solution to a biomedical or health information problem by applying computational and systems thinking, information science, and technology.
F5. Human factors and sociotechnical systems	Applying social behavioral theories and human factors engineering to the design and evaluation of information systems and technology.
F6. Social and behavioral aspects of health	Apply a model, which may be dependent upon the application area of the training program, to address a social and behavioral problem related to the health of individuals, populations, and organizations.
F7. Social, behavioral, and information science and technology applied to health	Integrate and apply the theories, models, and tools from social, business, human factors, behavioral, and information sciences and technologies to design, implement, and evaluate health informatics solutions.
F8. Professionalism	Demonstrate professional practices that incorporate ethical principles and values of the discipline.
F9. Interprofessional collaborative practice	Apply relationship-building skills and the principles of interprofessional communication in a responsive and responsible manner that supports a team approach to solve complex health and health information problems.
F10. Leadership	Employ leadership and fellowship methods, concepts, and tools to motivate others toward accomplishing a health informatics vision.

Academicians have also been discussing the integration of skills training into the health informatics curriculum for a long time. For example, new educational approaches related to emerging health information technologies were described, efforts to increase electronic health record (EHR) adoption were discussed, and hands-on exposure to health information systems during the graduate education was recommended to provide the necessary skills to solve interoperability issues [26]. Although regional, national, and independent accrediting agencies determine the core curriculum requirements for health informatics educational programs, these standards are not prescriptive. Health informatics faculty members who work in academic institutions, health informatics departments, and programs are expected to follow up on the changing requirements and update the content of their curriculum continuously. In addition, the Health Information Technology Workforce curriculum includes hands-on laboratory courses and encourages adding internship opportunities in the curriculum [27].

In addition, the IMIA’s working group encouraged the international health informatics community to begin a discussion on various big data and data training skills [28]. IMIA determined 3 domains and 12 learning outcomes that are related to data training and skills. These learning outcomes focus on health data management principles; structure and design principles of health records; principles of data representation and analysis; ethical and security issues; nomenclatures, vocabularies, terminologies, ontologies, and taxonomies; health administration and economics; basic informatics terminology; ability to communicate electronically; and methods of practical and theoretical informatics, mathematics, biometry, and epidemiology [29]. Although the digital divide is still a challenge, mobile broadband networks have reached 84% of the global population, and 46% of households have internet access around the world [30].

A number of health informatics students acquire skills training on the job rather than during their formal education, and recent studies emphasize the need for new models for skills acquisition [4,5]. However, the research on technology skills training in graduate health informatics curricula is still insufficient. The Office of the National Coordinator (ONC) for health informatics technology program recommended the integration of hands-on experience into the curriculum [27], but relatively few programs formally integrated digital technology skills training into their curriculum, and core technical skills to use digital technologies for medical apps were not well articulated in graduate health informatics and medical education programs [4].

According to the American Society for Training and Development, skills gaps in the organizations have been growing [31]. An EHR software called the Veterans Information Systems and Technology Architecture is the only hands-on training recommendation of the Workforce Development Program [27]. Although some nursing informatics programs have been integrating experiential learning in their graduate programs [32], most nursing schools provide limited technology training to teach how to enter, manage, and use data using various types of EHRs in traditional ways [33]. Similarly, most medical education programs limit technology-related training with the effective use of EHRs [34]. Conversely, an AMIA and AHIMA joint task force developed a detailed EHR core competencies matrix tool for different disciplines. This was one of the most important initiatives related to the development of EHR utilization skills in the clinical settings and was followed by similar initiatives [35].

Moreover, health informatics students need additional competencies to design and develop patient-centered health information systems, mine and analyze health care data, and use telemedicine and wireless remote monitoring systems. Evolving information technology and the growing number of medical devices and software apps for mobile devices require qualified workers with new skill sets, which were not included in the health informatics curriculum in the past. Overall, 5 employer-desired skill categories in bioinformatics—general, computational, biology, statistics and mathematics, and bioinformatics—were determined [36]. A recent report also emphasized health care organizations’ needs for analytics technology skills [37].

One of the major competency-based training initiatives was the Technology Informatics Guiding Education Reform (TIGER), which was established in 2006 to review informatics competencies for nursing students and practicing nurses. This initiative identified knowledge and skill set needs, which subsequently led to the development of an informatics competency framework for nurses that consists of basic computer skills, information literacy, and information management components. The TIGER Informatics Competencies Collaborative published their final report in 2009, and complex demands in health care led to the development of other national collaborative projects [38]. In addition, the Quality and Safety Education for Nurses Institute developed 6 competencies to provide safe and effective care, and one of them was focused explicitly on informatics skills to support clinical decision support and knowledge management care [39].

Despite several recommendations by professional organizations, a skills training framework for health informatics students is still not clearly defined. Existing skills training recommendations mostly focus on EHR training, and they generally do not include mHealth, home care, remote monitoring, AI, and data science training skills [4].

## Methods

### Study Design

A study to determine students’ confidence in specific health informatics skills was conducted. For this purpose, published health informatics competencies were evaluated by two researchers independently, and a questionnaire to investigate skill sets of graduate Master of Science (MS) in Health Informatics students was developed by surveying core facility directors [36], IMIA [29] and CAHIIM’s curriculum requirements [16,17], ONC for Health Information Technology Workforce Development Program’s recommendations [40], TIGER initiative’s final report [38], the Association of American Medical College report [31], and the Health Informatics Society of Australia’s health informatics skill recommendations [11]. To measure students’ specific software skills, the most widely used statistics and office app packages were selected.

The questionnaire was divided into three parts. Part 1 consisted of demographic questions. Part 2 collected information about self-assessed skill sets using Likert scale questions, and 24 health informatics skills were determined for the second part of the questionnaire. Part 3 explored students’ suggestions for a new curriculum using open-ended questions.

A Web-based questionnaire was sent to a total of 223 enrolled and graduated students in the master’s degree program. Overall, 45.7% (102/223) of the participants completed the questionnaire within 2 months of the survey period, and all survey submissions were suitable for analysis. Table 3 illustrates the general demographic characteristics of the participants.

**Table 3:** Frequency and percentage of respondents classified by demographic details (N=102).

General characteristics		Frequency, n (%)
**Gender**		
	Female	71 (69.6)
	Male	31 (30.4)
**Age (years)**		
	<34	68 (66.7)
	35-44	19 (18.6)
	45-54	12 (11.8)
	>55	2 (2.0)
**Enrollment status**		
	Currently enrolled	72 (70.6)
	Graduated	29 (28.4)
**Current occupation**		
	Information technology	24 (23.5)
	Clinical	29 (28.4)
	Health care medical services and products	21 (20.6)
	Other	16 (15.7)
	Not employed	11 (10.8)

### Questionnaire Validation

The questionnaire was tested on a small sample of respondents to identify problems with the construction and potential problems with the unclear wording. Face validity was established by an expert faculty member. The questionnaire was assessed, and the feedback about the clarity, friendliness of questions, and consistency was provided. Cronbach alpha was used to assess internal consistency, and it ranged from .9947 to .9952 (N=102). The overall reliability demonstrated excellent internal consistency.

### Participants and Data Collection

The inclusion criteria included the participants’ informed consent and being enrolled in or graduated from the MS in health informatics program at Adelphi University in Garden City, New York, United States. As skills training is not included in the curriculum, current students do not receive formal hands-on training. Therefore, all enrolled and graduated students were included in the study, and survey results were not divided.

An institutional review board–approved questionnaire was distributed to all graduated and enrolled students. The participants received the consent form and instructions to complete a Web-based questionnaire, and 4 reminder emails were sent at 1-week intervals. The survey was anonymous. Participation in the study was voluntary, and there was no grade or compensation.

## Results

### Quantitative Data Analysis

Among the respondents, 30.4% (31/102) were males, and 70.0% (71/102) were females. The largest percentage of respondents was aged less than 34 years; nearly one-third (31/102, 30.4%) of the participants were aged 35 to 54 years, and only 2 participants were older than 55 years. The majority of the respondents were currently enrolled in the program (73/102, 71.6%). Most of the respondents had an information technology– or health care–related occupation (74/102, 72.5%), and only 10.8% (11/102) of participants were not employed (Table 3).

### Identifying Student Confidence About Specific Health Informatics Skills

Benner’s 5-level model of skill acquisition framework (novice, advanced beginner, competent, proficient, and expert) was applied to assess students’ level of confidence [41]. Descriptive statistics were used to describe the students’ self-assessments of important skills in the forms of mean, standard deviation, and frequency. As health informatics accreditation competencies do not contain specific skill training recommendations and these components are not included in the current curriculum, graduate and enrolled students’ responses were analyzed together. There were 24 items, and the margin of error was determined as 7.16, assuming a 95% confidence level.

Respondents initially rated themselves higher on Microsoft Word essential skills. For skills to insert a table of contents, footnotes, endnotes, and cross-references, 84.3% (86/102) of respondents rated themselves as *extremely confident* or *very confident*, 9.8% (10/102) as *moderately confident*, and 6.9% (7/102) as *not at all confident* and *slightly confident*. The mean was 4.21 (expert).

Participants rated themselves as *proficient* in *Skills in evaluating health information systems* (mean 3.14), *Skills in training staff on system use, troubleshooting software and hardware issues* (mean 3.28), *Skills in performing math using Microsoft Excel and enter a calculation formula* (mean 3.81), *Skills in choosing evidence-based resources* (mean 3.75), and *Skills in compiling data from secondary sources* (mean 3.20). For advanced Microsoft Excel skills such as calculating sample variance and standard deviation, 45.1% (46/102) of participants rated themselves as *extremely confident* or *very confident*, 35.3% (36/102) as *moderately confident*, and 19.6% (20/102) *as not at all confident* and *slightly confident* (mean 3.48; Table 4).

Respondents rated themselves as *competent* in *Skills in programming mobile health informatics apps* (mean 2.24), *Skills in designing and leading health informatics projects* (mean 2.84), *Skills in setting up new businesses* (mean 2.61), *Skills in mining and analyzing data* (mean 3.00), *Skills in interpreting inferential statistics* (mean 2.63), *Skills in developing data visualization techniques* (mean 2.42), *Skills in using PICO to plan a search* (mean 2.50), *Skills in developing a database using Microsoft Access* (mean 2.82), *Skills in assessing data integrity and assessing data reliability* (mean 2.94), *Skills in evaluating the use of data capture technologies* (mean 2.82), *Skills in designing databases* (mean 2.34), *Skills in evaluating privacy and security infrastructure* (mean 2.34), *Skills in using Microsoft Word’s macro commands, creating dialog boxes, and understanding the notions of Visual Basic Application programming* (mean 2.62), *Skills in developing machine learning applications* (mean 2.30), *Skills in developing software to collect, organize, analyze, and interface with data* (mean 2.35), and *Skills in performing statistical tests using SPSS* (mean 2.74; Table 4).

For AI app development skills, 6.9% (7/102) of the participants rated themselves as *extremely confident* or *very confident*, 14.7% (15/102) as *moderately confident*, 79.4% (81/102) as *not at all confident* or *slightly confident*. The mean was 1.80 (advanced beginner; Table 4).

**Table 4:** Students’ responses regarding confidence with specific health informatics skills (N=102) (competency level according to Benner’s 5 levels of competencies: 0:00-1:00=novice; 1:0-2:00=advanced beginner; 2:0-3:00=competent; 3:01-4:00=proficient; 4:01-5:00=expert).

Survey item	Value, mean (SD)	Extremely confident/very confident, n (%)	Moderately confident, n (%)	Not at all confident/slightly confident, n (%)	Internal reliability Cronbach alpha	Interpretation
Skills in evaluating health information systems and preparing recommendations to improve functionality	3.14 (1.12)	40 (39.2)	31 (30.4)	31 (30.4)	.9947	Proficient
Skills in developing machine learning apps for personalized health monitoring	2.30 (1.07)	13 (12.7)	31 (30.4)	58 (56.8)	.9948	Competent
Skills in building interfaces and developing and programming mobile health informatics apps	2.24 (1.06)	10 (9.8)	31 (30.4)	61 (59.8)	.9948	Competent
Skills in setting up new businesses and entrepreneurship	2.61 (1.21)	25 (24.5)	21 (20.6)	56 (54.9)	.9948	Competent
Skills in training staff on system use and troubleshooting software and hardware issues	3.28 (1.27)	47 (46.0)	25 (24.5)	30 (29.4)	.9947	Proficient
Skills in mining and analyzing data	3.00 (1.08)	34 (33.3)	31 (30.4)	37 (36.3)	.9948	Competent
Skills in interpreting inferential statistics	2.63 (1.04)	20 (19.6)	29 (28.4)	52 (51.0)	.9948	Competent
Skills in developing software to collect, organize, analyze, and interface with data	2.35 (1.10)	15 (14.7)	29 (28.4)	58 (56.8)	.9948	Competent
Skills in developing artificial intelligence apps	1.80 (1.02)	7 (6.9)	14 (13.7)	81 (79.4)	.9951	Advanced beginner
Skills in developing data visualization techniques	2.42 (1.16)	19 (18.6)	25 (24.5)	58 (56.8)	.9948	Competent
Skills in designing and leading health informatics projects	2.84 (1.15)	28 (27.5)	36 (35.3)	38 (37.3)	.9947	Competent
Skills in assessing data integrity and assessing data reliability	2.94 (1.10)	35 (34.3)	29 (28.4)	38 (37.3)	.9948	Competent
Skills in compiling data from secondary sources	3.20 (1.09)	43 (42.2)	34 (33.3)	25 (24.5)	.9948	Proficient
Skills in evaluating the use of data capture technologies	2.82 (1.01)	24 (23.5)	37 (36.3)	41 (40.2)	.9949	Competent
Skills in designing and developing databases	2.34 (1.10)	15 (14.7)	27 (26.4)	60 (58.8)	.9949	Competent
Skills in evaluating privacy and security infrastructure	2.34 (1.11)	18 (17.6)	28 (27.5)	56 (54.9)	.9948	Competent
Skills in using Microsoft Word to insert a table of contents, footnotes, endnotes, and cross-references	4.21 (0.92)	85 (83.3)	10 (9.8)	7 (6.9)	.9952	Expert
Skills in using Microsoft Word’s macro commands, creating dialogue boxes, and understanding the notions of Visual Basic Application programming	2.62 (1.23)	24 (23.5)	32 (31.4)	46 (45.1)	.9948	Competent
Skills in developing a database using Microsoft Access	2.82 (1.09)	31 (30.4)	23 (22.5)	48 (47.1)	.9948	Competent
Skills in performing math using Microsoft Excel and enter a calculation formula	3.81 (1.03)	62 (60.1)	27 (26.5)	13 (12.7)	.9949	Proficient
Skills in using Microsoft Excel for statistics such as calculating sample variance and standard deviation	3.48 (1.10)	46 (45.1)	36 (35.3)	20 (19.6)	.9948	Proficient
Skills in performing statistical tests using SPSS	2.74 (1.21)	28 (27.5)	30 (29.4)	44 (43.1)	.9947	Competent
Skills in choosing evidence-based resources	3.75 (0.99)	66 (64.7)	24 (23.5)	12 (11.8)	.9949	Proficient
Skills in using PICO to plan a search	2.50 (1.19)	21 (20.6)	32 (31.4)	49 (48.0)	.9948	Competent

### Qualitative Data Analysis

The qualitative data analysis process to identify patterns and themes was inspired by Braun and Clark’s thematic analysis method [42]. The 6 steps of thematic analysis were used, and 4 themes emerged from the data:

- Theme 1: EHR software training: Participants expressed an interest in hands-on training in EHR documentation and security (Textbox 1).
- Theme 2: Data science, visualization, and analytics: Respondents expressed a strong preference for hands-on experience with Structured Query Language (SQL), Tableau, Crystal Reports, and other database and data visualization products (Textbox 1).
- Theme 3: Software training and app development: Students emphasized the need for programming classes and coding skills and requested courses that focus on entry-level programming, HTML courses, and Microsoft Project (Textbox 1).
- Theme 4: Specialization courses: Participants acknowledged a desire to receive certifications and indicated the need for specific tracks depending on career plans (Textbox 1).

**Students’ course requests.**

**Theme 1: Electronic health record software training**

“There could be more exposure to and training on information technology that we will come into contact with in the field like the EHR.”

“Maybe there can be a class on EMRs which can incorporate what is out right now and teach students about what makes a health care system successful and lasting.”

“If one class required the students to virtually build a system.”

“Hands-on working of top EMR like EPIC.”

“I think medical terminologies could be added to the curriculum. I also think the program could offer different tracks so we could choose.”

“Having access to an EMR system and being able to utilize it.”

“Perhaps purchasing a low-cost small practice EHR and over the course of the semester have students learn the backend; how to create users, manage security, edit forms, notes, and templates. Divide the class into groups assign a new functionality to be built within the system (anew note for example) task the team with building that item including everything from building a project plan to creating training materials the rest of the class on the new functionality.”

**Theme 2: Data science, visualization, and analytics**

“Interactive training for VBA, Tableau, Python.”

“One change would be to definitely increase the actual use of apps such as the Microsoft suite (Excel, Word, Access, Project, Visio), as well as learning more about Structured Query Language (SQL). Database creation and querying are such important functions in IT.”

“More on reporting data.”

“Data modeling and visualization classes based on industry software.”

“A little more database work and knowledge could help.”

“More technical courses—data analytics, predictive models, cognitive computing, Crystal Reports.”

“More exposure to databases and SQL, or coding of some kind.”

“During my journey I have learned many technical staff such as database design and management, health care information management, security design and other similar subjects, but in my opinion the program should include more practical technical staff like teaching a programming language.”

“I wish there was more courses that was geared toward MS Excel and PowerPoint. Being sufficiently prepared with these apps can build confidence and adequately prepare an individual for employment.”

“Add technical skills; SQL, Java, ...”

“I wish we learned SQL.”

“SQL class.”

**Theme 3: Software training and app development**

“I can’t stress enough the need for a programming class to be added to the curriculum. Since graduating, I have had to invest in this training as it is needed when building reports.”

“To add more classes that involve direct software learning as opposed to just researching and writing papers. It would have definitely helped me in the future.”

“Include more IT related classes (programming, software development) that will actually help in our career paths. Classes should be similar to what health Informaticians will face practically in the workforce rather than textbook-based.”

“One suggestion that I would give to add to the existing curriculum is incorporating more informatics and technology. Let’s say coding for example. Although we had the opportunity to understand how to analyze data I do feel like in terms of technology there are a lot more components to learn.”

“Teaching basic coding skills/HTML, allowing hands-on experience identifying system issues or software bugs, would have been very helpful to have more technical experience.”

“Possibly video lectures or some step by step instructions on developing software, databases, computer programming, etc.”

“Entry level programming.”

“Additional use of Microsoft Project.”

“I would suggest incorporating more skills-based courses in the program. Skills that frequently seen in the field of health care informatics. A lot of the students that I took the classes with did not have clinical backgrounds. As a clinical informaticist, it’s imperative to understand the clinical side of the health care industry. It would be very beneficial to future students to receive some type of course or resource that includes that.”

“I wish some of the classes included real-life systems and applications we could practice more with. It just seemed like a lot of material to cover in a short amount of time.”

“The MSc online program should shift focus from theory to more practice beyond preceptorship. Employers want staff who have hands-on experience in various software and systems.”

**Theme 4: Specialization courses**

“You need to add all of the focused subject areas. Students should be able to specialize in the last few courses. Some of the classes are not useful depending on the field of interest.”

“I even wonder if adding specific tracks would be a good idea. While some students may want more project management courses, others may prefer database querying and reporting, or research, or even security. Perhaps specified “minors,” so to speak, could help students gain more knowledge in their areas of interest and make them more confident in applying for specific jobs after graduation.”

“I believe that it’s very important that the Practicum allows for hands-on experience with the actual hardware set-up so that the student will learn how to troubleshoot a technical issue. I have not yet found a job in the field, and one of my biggest fear is that I lack the technical experience. Besides the jobs I have seen, are seeking applicants with years of experience. If the Practicums entail just as much learning experience with the hardware as well as the software, that would certainly be advantageous.”

“Instead of a practicum, perhaps the program could offer other options that are often associated with different career paths that a degree in health care informatics could take. For instance, the program could offer the option of getting certified as a Project Management Professional (PMP), or certified in Data Analytics, etc. These options would boost a resume significantly and are directly related to potential career options within the realm of health care informatics. I understand job placement is a challenging undertaking for universities. However, this could be a great option to make sure your students have an advantage in the hiring process.”

## Discussion

### Principal Findings

Even though the demand for health informatics graduates has been changing, to the best of our knowledge, the number of studies that focus on hands-on health informatics skills training is limited. Recently, the Institute of Education Sciences developed a classification system called the Integrated Postsecondary Education Data System to track and report the fields of study [43]. However, this classification system does not cover all potential career paths as the professions related to health informatics fall under several occupations, and therefore, it is quite challenging to define health informatics career trends. Another recent study analyzed the content of US health care data scientist job postings to identify the required qualifications and skills for data scientist positions and emphasized the need for higher levels of education and skills training needed for health care data scientists [3].

In this study, we evaluated students’ perceived skills and self-confidence to develop health informatics apps. As professional and accrediting organizations have not determined distinct boundaries between competency and skill terms, we used these terms interchangeably. Currently, formal health informatics skills training with medical devices and apps are limited. Although students without any health informatics education might become an expert in programming, developing, and using an innovative health informatics app or system, others with formal education might not have any hands-on skills using the same apps. Hence, using competency and hands-on skills interchangeably in all cases is quite challenging. This research revealed that most students were employed (91/102, 89.2%), and presumably, they were knowledgeable about the required skills. Participants did not consider themselves experts in any skills, which indicates the need for the integration of skill-based training into the health informatics curriculum, except for skills in using Microsoft Word’s macro commands.

Furthermore, our research has several implications. First, this analysis identified a gap between existing competencies and in-demand skills. Developing innovative solutions to improve health care quality has become the major focus of leading health informatics companies, and recent publications emphasize that tomorrow’s workforce needs to design, develop, and implement innovative systems and work with new medical devices, patient monitoring apps, telemedicine, and smart home systems [5]. Owing to the lack of formal skills training, most health informaticians gain these practical experiences during their employment; thus, employers have been launching upskilling initiatives to keep their company competitive [44].

Second, this study revealed the need to determine new occupation-specific health informatics terms that will define different levels of practical know-how to generate disruptive ideas and design, develop, and implement sophisticated innovative technological solutions to health problems.

Third, we also identified the need to develop a specific competency assessment framework. Currently, AMIA uses Miller’s competency framework, which was initially developed to assess the clinical competence of medical school graduates. The Miller’s pyramid consists of four levels of clinical competence: knowing signs and symptoms (knows), knowing how to utilize exam and laboratory test data to diagnose a disease (knows how), demonstrate clinical performance (shows how), and being able to apply knowledge into practice (does) [45]. Although the Miller’s pyramid is widely accepted in medical practice to assess clinical competence, its application to health informatics has some limitations as this assessment model was not designed to assess any informatics competencies. As health informatics is an interdisciplinary field, the graduates might work in a wide range of settings and can follow different career paths, which makes the development of the competency framework extremely complex.

**Figure 1:**
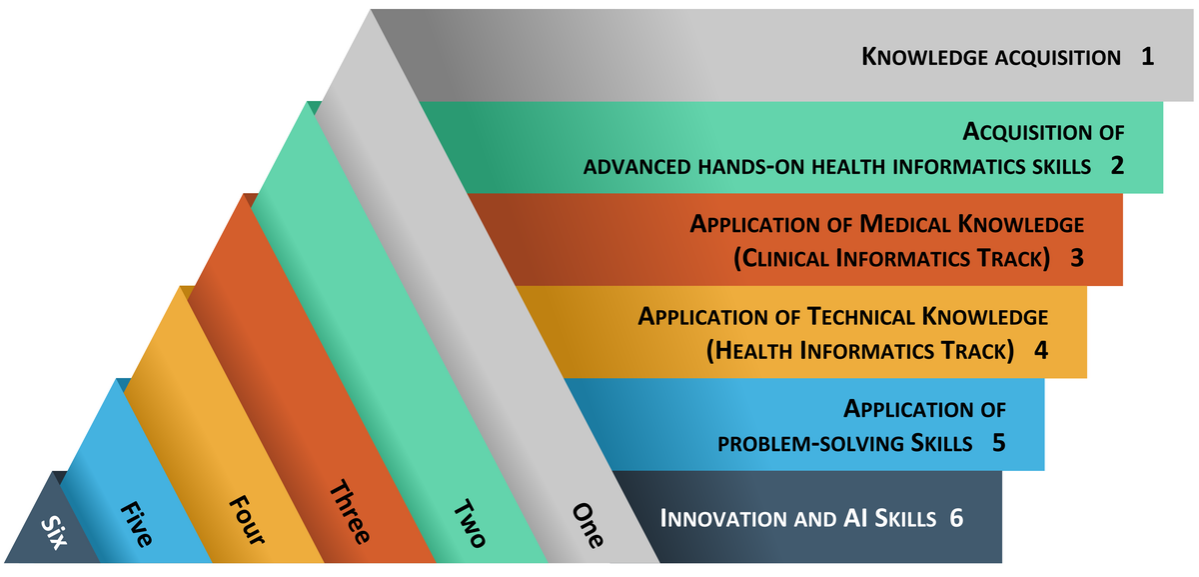
Proposed health informatics competency framework. AI: artificial intelligence.

We developed a new framework that will include different tiers for evolving hands-on health informatics competencies (Figure 1). This competency framework divides the development of practical health informatics competencies into 6 hierarchical processes. The pyramid starts with *knowledge acquisition* at the bottom level. The next competence level is achieved when students acquire *advanced hands-on health informatics skills* to use specific computer software programs, sensor-based decision support systems, and other sophisticated patient monitoring apps. The third and fourth tiers represent applications of medical knowledge and technical knowledge. Although all health informaticians need to become familiar with these two competencies, teaching clinical health informatics tracks concentrating on the application of medical knowledge using health informatics systems and teaching nonclinical tracks concentrating on the application of technical knowledge such as programming, application of algorithmic principles, design, and development of mobile apps and other data science skills that we mentioned in our study might have more profound and meaningful outcomes. The fifth tier focuses on the application of problem-solving skills to manage and administer health informatics apps and programs. Finally, the sixth tier concentrates on innovative skills. Although most health informatics programs include capstone courses, these courses are usually designed to apply the knowledge gained through the master’s degree program rather than teaching new skill sets.

**Figure 2:**
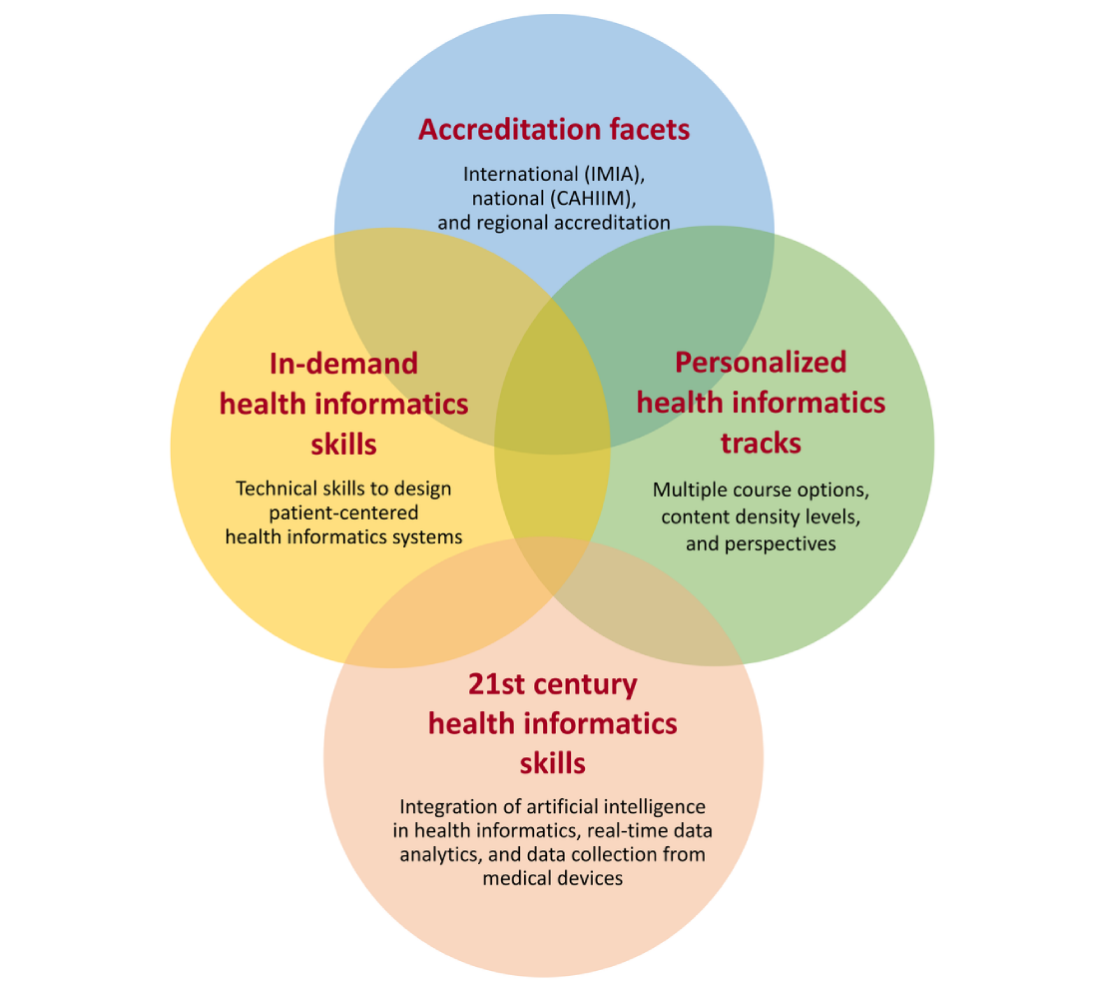
Enhanced health informatics curriculum components. CAHIIM: Commission on Accreditation for Health Informatics and Information Management Education; IMIA: International Medical Informatics Association.

Health informatics specialists should know how to analyze and interpret health care data and identify potential areas of applications of AI. Defective AI algorithms can cause severe and unforeseen health consequences. However, integration of experiential AI training in health informatics curriculum and determination of necessary skill sets for different specializations is quite a challenging task as AI technology has many components such as machine learning, deep learning, pattern recognition, real-time data analytics, model building, data collection, and data visualization. This research demonstrated that participants defined themselves as an advanced beginner for skills in developing AI apps. This definition could be considered as being insufficient; however, a master’s degree program should consider students’ career perspectives and provide individualized tracks in addition to meeting mandatory accreditation standards. We propose that specific health informatics skills training should be identified using the enhanced health informatics curriculum components described in Figure 2 and be updated on a yearly basis.

Including R, Python, inferential and descriptive statistics, machine learning, database systems, and SQL, data presentation and visual encoding courses in the curriculum without real-life medical apps might not be enough to provide the required skills as students need to learn how to operate sophisticated medical equipment and remote monitoring devices and solve interoperability challenges. For example, with the hands-on laboratory exercises, students will be able to develop clinical decision support apps that can collect data from a wireless blood pressure monitor, wireless blood glucose meters, digital weight scales, and write the program code to integrate these apps with other databases. Consequently, depending on the students’ career plans, they might need further specialization such as integrating machine learning code with sophisticated medical software. Recent studies demonstrate the effectiveness of hands-on health informatics skills exercises [5,39].

### Limitations

Several limitations need to be acknowledged. This research was conducted in an academic institution, and the feedback was limited to the MS in health informatics students’ assessments. All students with different educational backgrounds were included in the study and analyzed together because skill-based training is not a part of the current curriculum. Thus, conducting regular national and international studies to analyze students’ confidence levels and course requests and comparing responses of students within the same educational backgrounds would be helpful.

### Conclusions

**Figure 3:**
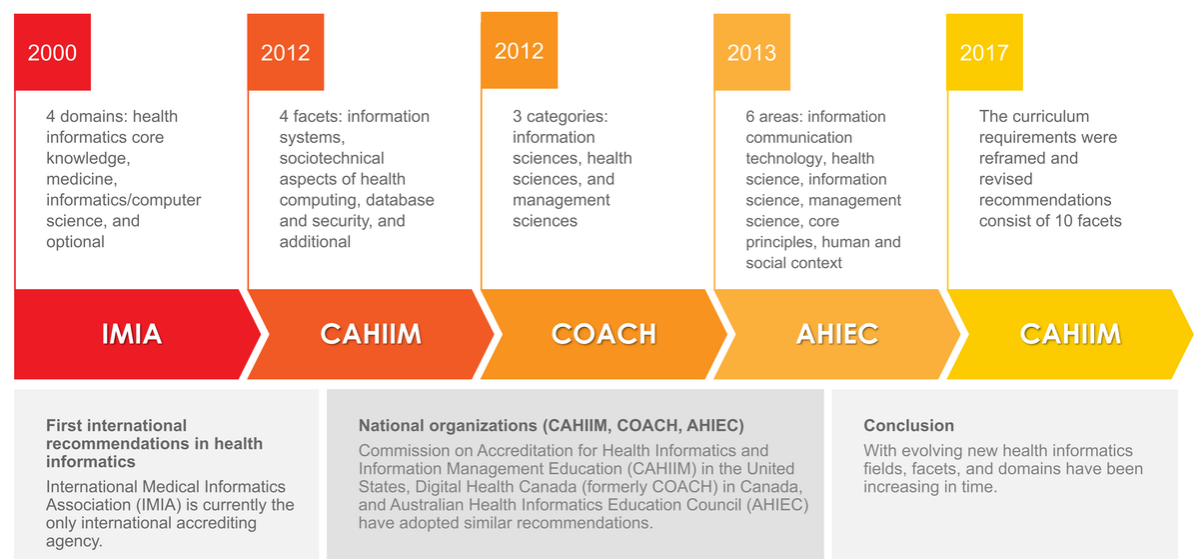
Evolving health informatics curriculum competencies. AHIEC: Australian Health Informatics Education Council; CAHIIM: Commission on Accreditation for Health Informatics and Information Management Education; IMIA: International Medical Informatics Association.

The main objective of this study was to highlight evolving health informatics competencies rather than provide detailed information about country-level competencies. Owing to the universal nature of technology, health informaticians use the same data standards, methods, and algorithms to store, retrieve, and analyze the data around the world. Although national and international organizations have determined different foundational domains, professional and accrediting organizations have been updating their recommendations frequently and adopting similar measurable competencies (Figure 3) [13]. For instance, IMIA’s updated educational recommendations for nursing informatics and health informatics are the same [46]; conversely, the current studies emphasize the need for customization. We also observed that the existing literature and curriculum recommendations did not clearly delineate the difference between undergraduate and graduate health informatics competencies. As mentioned earlier, even though some recent publications assess health informatics training and identify universal competencies, there are still limited studies about skills training in the graduate health informatics curricula [47,48].

Health informatics graduates need hands-on experience with various health informatics tools and apps to develop skills and the ability to apply this practical expertise to unfamiliar situations, serve as subject-matter experts, and lead and manage innovative projects. Regional, national, and international accreditation standards, and in-demand technical skills to use and develop patient-centered health informatics systems could be taken into consideration when determining health informatics curriculum components. It is also essential to capture students’ perspectives before developing skills training components and to develop an up-to-date health informatics skills training framework depending on different medical specialties and health care needs for physicians, nurses, pharmacists, and medical and laboratory technologists. Developing new terminologies that will clearly specify the difference between competency-based and skill-based approaches for each health informatics discipline might be useful. Further studies that evaluate employers’ feedback and students’ perceptions after they get hired are suggested to determine the potential gaps and needs in health informatics skills training.

## Abbreviations

AHIMA: American Health Information Management Association
AI: artificial intelligence
AMIA: American Medical Informatics Association
CAHIIM: Commission on Accreditation for Health Informatics and Information Management Education
eHealth: electronic health
EHR: electronic health record
EU: European Union
ICT: information and communication technology
IMIA: International Medical Informatics Association
mHealth: mobile health
MS: Master of Science
ONC: Office of the National Coordinator
SQL: Structured Query Language
TIGER: Technology Informatics Guiding Education Reform
